# A community programme to reduce salt intake and blood pressure in Ghana [ISRCTN88789643]

**DOI:** 10.1186/1471-2458-6-13

**Published:** 2006-01-24

**Authors:** Francesco P Cappuccio, Sally M Kerry, Frank B Micah, Jacob Plange-Rhule, John B Eastwood

**Affiliations:** 1 Clinical Sciences Research Institute, Warwick Medical School, UHCW Campus, Clifford Bridge Road, Coventry CV2 2DX, UK; 2 Division of Community Health Sciences, St George's University of London, SW17 0RE, UK; 3 Komfo Anokye Teaching Hospital, Kumasi, Ghana; 4 Division of Cellular & Molecular Medicine, St George's University of London, SW17 0RE, UK

## Abstract

**Background:**

In Africa hypertension is common and stroke is increasing. Detection, treatment and control of high blood pressure (BP) is limited. BP can be lowered by reducing salt intake. In Africa salt is added to the food by the consumer, as processed food is rare. A population-wide approach with programmes based on health education and promotion is thus possible.

**Methods:**

We carried out a community-based cluster randomised trial of health promotion in 1,013 participants from 12 villages (628 women, 481 rural dwellers); mean age 55 years to reduce salt intake and BP. Average BP was 125/74 mmHg and urinary sodium (UNa) 101 mmol/day. A health promotion intervention was provided over 6 months to all villages. Assessments were made at 3 and 6 months. Primary end-points were urinary sodium excretion and BP levels.

**Results:**

There was a significant positive relationship between salt intake and both systolic (2.17 mmHg [95% CI 0.44 to 3.91] per 50 mmol of UNa per day, p < 0.001) and diastolic BP (1.10 mmHg [0.08 to 1.94], p < 0.001) at baseline. At six months the intervention group showed a reduction in systolic (2.54 mmHg [-1.45 to 6.54]) and diastolic (3.95 mmHg [0.78 to 7.11], p = 0.015) BP when compared to control. There was no significant change in UNa. Smaller villages showed greater reductions in UNa than larger villages (p = 0.042). Irrespective of randomisation, there was a consistent and significant relationship between change in UNa and change in systolic BP, when adjusted for confounders. A difference in 24-hour UNa of 50 mmol was associated with a lower systolic BP of 2.12 mmHg (1.03 to 3.21) at 3 months and 1.34 mmHg (0.08 to 2.60) at 6 months (both p < 0.001).

**Conclusion:**

In West Africa the lower the salt intake, the lower the BP. It would appear that a reduction in the average salt intake in the whole community may lead to a small but significant reduction in population systolic BP.

## Background

Non-communicable diseases are an important threat to the health of adults in Africa [1]. Worldwide, stroke is second only to ischaemic heart disease as a cause of death (over 4 million in 1990), and most of these deaths are in developing countries [2,3]. In sub-Saharan Africa, hypertension is common [4-7] and its detection, prevention, management and control should now be regarded as a priority [8]. It is estimated that if the 10–20 million people believed to have hypertension in sub-Saharan Africa were treated, about a quarter of a million deaths and twice as many long-term disabilities would be prevented annually [8]. Furthermore, in Africa, the reduction in population attributable risk when blood pressure (BP) is lowered is 13 times greater than in the USA [8]. However, where health-care provision is poor, detection and management of hypertension can only be haphazard and unreliable. Population-wide strategies to reduce BP could have an important impact on the number of strokes in the community. Whilst there is some data from Ghana using hospital series that stroke rates are increasing [9-11], one of the general problems in sub-Saharan Africa is the lack of reliable population-based morbidity and mortality data on stroke and other chronic diseases. Death certification and causes of death are not consistently recorded and cause-specific mortality rates are derived through verbal autopsies. Recent data from Tanzania [12] and South Africa [13] suggest a high burden of stroke, comparable to those seen in developed countries.

The World Health Organization regards population-wide strategies as an integral part of the overall approach to the prevention of cardiovascular disease worldwide, especially those that include dietary modification [14,15]. There is good evidence that a reduction in salt intake reduces BP [16] and that people of black African origin living in Africa respond well [17-19]. In the western world, it is very difficult to implement successful salt reduction strategies in the population since most of the salt ingested is in processed food. So, any intervention would involve the participation of the food industry and some attempts have been made in Portugal [20] and Finland [21]. In contrast, in populations whose intake of processed food is negligible – such as are found in many areas of sub-Saharan Africa – salt reduction strategies should be relatively easy to implement and have a good chance of success. To date, however, there have been no such community intervention studies in sub-Saharan Africa so the feasibility and effectiveness of a health education approach to BP reduction is unknown.

We started a programme with a view to establishing the feasibility of salt reduction as a way of reducing BP in twelve rural and semi-urban villages in the Ashanti region of Ghana where we suspect that hypertensive-related vascular diseases (namely stroke, renal and heart failure) account for a significant proportion of morbidity and mortality [22]. We conducted a pilot study, a household survey and population census, a survey of risk factors, subsequently randomised villages into clusters to receive general health education or more specific education as to how to reduce salt intake, then followed up the participants for six months to evaluate the effect on sodium excretion and BP. This paper describes the whole programme, highlights the strengths and limitations of our approach as well as the lessons learnt from our work, and suggests an interpretation in the context of the low-resource countries of sub-Saharan Africa.

## Methods

### Ethics

The study protocol was approved by the Committee on Human Research Publication and Ethics of the School of Medical Sciences, Kwame Nkrumah University of Science and Technology, Kumasi and endorsed by the St George's Healthcare NHS Trust Local Ethics Committee. As the study got under way meetings were held with the Chief and the Council of Elders in each village to explain the study and to seek their permission for the study to take place in their village.

### Study area and population

The study was undertaken in the Ashanti region of central Ghana where the Disability Adjusted Life Expectancy (DALE) is 45.5 years [23]. Our study encompasses 12 communities in the Ejisu-Juabeng and Kumasi Districts [24]. The villages were chosen in consultation with local health workers [24]. The villages had little day-to-day contact and direct communication with each other [24], and transport between the villages is not generally available. Six villages were considered to be rural as they lack electricity and piped water, have a small population and are some distance from Kumasi [24,25]. The main occupation is farming. The remaining six villages were considered semi-urban. They are closer to Kumasi and usually have electricity and piped water [24,25]. Although the main activity is farming, trading is a frequent occupation. The Komfo Anokye Teaching Hospital, the only Tertiary Referral Hospital in the region, serves the area. In 1995 the hospital set up a BP clinic that now sees around 500 new patients a year [22].

### Household survey and population census

Following agreement with the Chiefs and Elders in each village, a team of fieldworkers, fluent in both English and Twi (the local language), carried out a population census between January and March 2001 in order to create age-and-sex registers for the villages [24]. A total of 16,965 individuals (6,597 rural and 10,368 semi-urban) from 1,460 households (750 rural and 710 semi-urban) were identified. The 12 villages had an average population (all ages) of 1,414 inhabitants (range 562–1,966) [24]. The adult population (>16 years) was 50% in rural and 57% in semi-urban villages. The population structure of these villages was comparable to that reported for Ghana as a whole. There were 2,743 adults aged 40–75 years.

### Recruitment of participants

Between 95 and 250 subjects aged 40 to 75 from each village, total 1,896, were selected by stratified random sampling by age and sex from the census of all inhabitants in the village so that the total sample selected matched the overall population structure. Between June 2001 and June 2002 a multidisciplinary study team set up a data collection centre in each village. All members of the team had been trained to take measurements and to carry out procedures while adhering to a protocol. The information sheet and consent form (available in both English and Twi) was explained to each participant who, if agreeing to take part, gave consent either by signing their name or applying a thumbprint.

### Baseline assessment

For each consenting participant a fieldworker completed a detailed questionnaire, and measured height, weight and BP. Where necessary the questioning was in Twi after local verification of common expressions and translations. Socio-economic, lifestyle and medical information was collected. Information was also gathered on five salty foods – koobi, momoni, kako (all salted fish), salted pigs' feet and salted beef [25]. The respondents were asked if they ate these foods regularly and whether they added salt at the table or during cooking.

Height and weight were measured and body mass index (BMI) calculated [6]. BP and pulse rate were measured, and participants were also asked whether they were on regular anti-hypertensive drug therapy [6]. Venous blood was collected using vacutainers. Samples were transported to Komfo Anokye Teaching Hospital for aliquoting and storage [6]. Two consecutive timed 24-hour urine samples were obtained from each participant under close supervision. The urine samples were transported the same day to Komfo Anokye Teaching Hospital. Aliquots were prepared and frozen at -20°C. Serum and urine were later shipped to London on dry ice for long-term storage.

### Randomisation and power calculations

**Figure 1:**
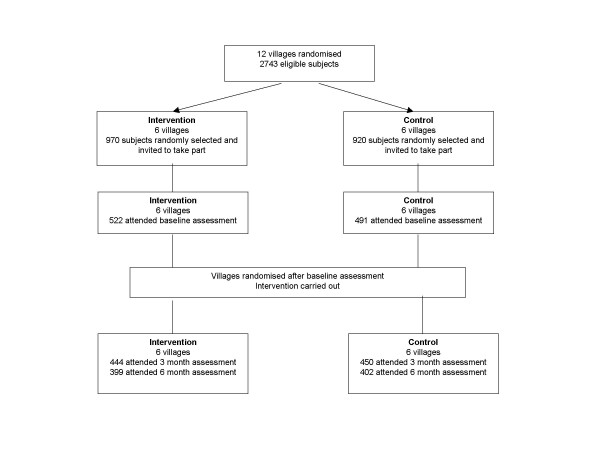
Flow chart of the trial.

After recruitment of participants, villages were allocated to intervention and control groups (Figure 1). Villages were randomised in blocks of two, and stratified for locality (semi-urban or rural) by an independent statistician [26]. Thus, intervention could begin in the first two villages while subjects in the second two were being recruited. The main outcomes of the trial were change in 24 h urinary sodium over the follow-up period and change in systolic BP. An average of 70 participants completing the trial from each village would allow a change in systolic BP of 4.8 mmHg to be detected with 90% power, using a 5% significance level, assuming the standard deviation of the change in BP to be 12.6 mmHg and the intra cluster correlation coefficient to be 0.02 [26]. Initially, 95 subjects were invited from each village to allow for a 25% refusal and attrition rate. As the response rate in the first four villages was lower than anticipated, more subjects were recruited in the remaining villages to obtain sufficient subjects overall and to allow for a greater refusal rate. The groups were comparable for baseline characteristics (Table 1).

### Intervention programme

An intensive health education programme was carried out by community health workers. The educational and health promotion sessions were open to all villagers, irrespective of their participation into the trial. They were encouraged to attend by preliminary meetings with health visitors, the endorsement of their chief and community leaders. The meetings were held daily for the first week of the study and once weekly thereafter. The sessions were held in communal areas like churches, churchyards, schools, community centres. The sessions lasted approximately one hour (both for intervention and control). We did not record the actual number of attendants at each meeting as they were in relatively large numbers and, therefore, we are unable to report response rates at each session. The spirit of the intervention was to expose the whole community to the health promotion message and to test only a random proportion of them. A standard health education package from the Ghana Ministry of Health was used in all the villages. Flip charts were the main medium of communication; they consisted of double-sided A3-sized sheets with a colour picture on one side (shown to the participants) and written text on the other (facing the health visitor and used as a prompt). The public health issues included prevention of malaria, infective diarrhoeas and roundworm infection, as well as enhancing awareness of diabetes and high blood pressure. No mention was made of any possible dietary prevention of hypertension. In the intervention villages additional advice was given not to add salt to food and in cooking, to limit the amount of koobi, momoni, kako and tilapia (salted fish), salted pigs' feet and salted beef and to soak the items in water overnight before eating them. In order to avoid potential bias and contamination, we sought to maintain 'blindness' of the participants as to which dietary intervention they were receiving. On the other hand, we could not keep the community health nursing staff blind to the main objectives of the study. The feasibility of this approach had been tested in a small pilot study reported elsewhere [17].

Participants randomised to take part into the trial (both intervention and control) were given a small gift at the end of the study consisting of soap, rice, sugar, tinned mackerel, paracetamol, folic acid and some vitamin supplements; however they did not know this prior to participating, so that the response rate is likely to represent an unbiased response to a health promotion initiative. Likewise, the whole village was presented with a gift at the end of the study consisting of either a television set for the communal room (where there was electricity) or corrugated roofing sheets as a contribution to the building of local schools or community areas.

### Follow-up

Participants were re-examined at 3 months and 6 months. At the 3-month visit a history of drug therapy was taken, and weight, BP and pulse were measured and recorded. Two 24-hour urine samples were collected but blood samples were not taken. At 6 months the same measurements were made, and on this occasion a blood sample as well. These urine and blood samples were processed in a similar way to the baseline samples. At 3 months 894 subjects attended and 889 (558 women and 331 men) were included in the analysis of urinary sodium. Of these 833 (531 women and 331 men) were based on two samples and 56 on one sample because a blinded assessment of the data indicated that one sample was incomplete or over-collected; 9% of men and 5% of women had only one sample. At 6 months 801 subjects attended and 800 (493 women and 307 men) were included in the analysis of urinary sodium. Of these 754 (465 women and 289 men) were based on two samples and 46 on one sample; 4% of men and 6% of women had only one sample. The overall response rate at baseline was 53% (1,013/1,896) but this varied considerably between villages (range 40% to 88%). Response rates were higher in women than men; 58% (628/1,088) v 48% (385/808) and responders were 2.0 (95% CI: 0.8 to 3.1) years older than non responders. In rural villages men and women responded similarly (57% v 60%; difference -3% [-21 to 20%]) but in the semi-urban villages men were less likely to respond (41% v 56%; difference -15% [26 to -4%]). Follow-up rate at 6 months was similar in men and women (307/385 or 80% vs 494/628 or 79%). Attenders were 2.5 (0.8 to 4.2) years older than those who dropped out. Follow-up rates varied between villages from 62% to 92%.

### Statistical analysis

Regression analysis with robust standard errors was used to assess the effects of time of day on BP and pulse rate using the baseline measurements, adjusting for age, sex, locality, BMI and interaction between age and sex. All BP and pulse rate measurements were adjusted using these models prior to further analysis. The relationship between BP and sodium excretion, and between change in BP and change in sodium excretion were estimated using a random effects regression model with maximum likelihood estimation to allow for clustering within villages. The intervention effect was estimated as the difference in the change in the outcome in the intervention and control groups so that a negative difference favours the intervention. The analysis was carried out using a random effects regression. Adjusted changes in urine sodium-to-creatinine ratio for each village were estimated from ordinary least squares regression in each village and plotted using a random effect meta-analysis procedure. The correlation between the adjusted change in BP and the number of subjects was calculated using the meta-regression procedure. All analyses were carried out using Stata version 7.0.

## Results

### General characteristics

There were 1,013 participants in the study (385 men and 628 women); of these 532 were from semi-urban and 481 from rural villages. Ninety per cent of them reported being aged 40 to 75 years (mean age 54.7 years [SD 11.3]). Salt was almost invariably added to food in cooking (98%) and salted foods such as fish and meat were eaten in both rural and semi-urban villages. Salt was often added at the table (52%), more often in rural villages than in semi-urban settings (59 v 45%; p < 0.01), although the total salt consumed as measured by urinary sodium was similar (99 v 103 mmol/day). Urinary potassium levels were higher in rural villages (58 v 40 mmol/day; difference 18 mmol/day [11 to 26]; p < 0.001). In semi-urban villages people were heavier (BMI 22.3 [4.6] vs 19.8 [3.2] kg/m2, p < 0.001) and had higher BP (129/76 [26/14] vs 121/72 [25/13] mmHg, p < 0.001 for both systolic and diastolic BP). The prevalence of hypertension increased with age and was more common in semi-urban settings [6].

### Effect of time of day

There was a significant difference in the time of the day at which BP and pulse rate were taken at baseline (mean time of the day 9.28 [SD 1.39] equivalent to 9:17 am) compared with the two follow-up periods (7.57 [0.77] equivalent to 7:34 am at 3 months and 7.67 [0.73] equivalent to 7:40 am at 6 months), due to the necessity for taking much larger numbers of measurements at baseline. This difference in time of day of screening was associated with a significant drift in both BP and pulse rate. At baseline, there was a 1.90 mmHg [95% CI 0.63 to 3.24; p < 0.001) lower systolic and a 1.45 mmHg [0.66 to 2.20; p < 0.001) lower diastolic BP for every hour of time of day of screening with a higher pulse rate of 0.85 bpm per hour (0.14 to 1.56; p < 0.001). These estimates are compatible with those reported in earlier studies both in whites [27] and black Africans [28]. Further analyses are therefore reported with BP and pulse rate adjusted for time of day and standardised to 8:00 am.

### Relationship between urinary sodium and BP

There was a significant and positive relationship between the level of salt intake (however estimated) and both systolic and diastolic BP (Table 1). When allowing for the potential confounding effects of age, gender, locality, BMI and clustering, there was a 2.2 mmHg lower systolic and a 1.0 mmHg lower diastolic BP for a 50 mmol/day lower urinary sodium excretion. These relationships were significant also when expressed as sodium-to-creatinine ratio and sodium-to-potassium ratio (Table 1).

### Effect of the intervention programme on salt intake

**Figure 2:**
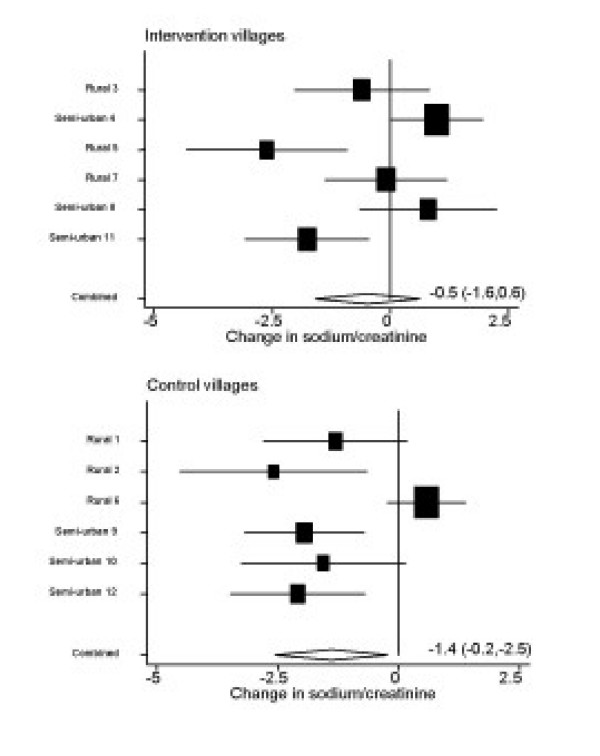
Changes in sodium-to-creatinine ratio adjusted for age, sex, BMI at baseline and locality (rural or semi-urban) in each village for the intervention and control groups of villages.

**Figure 3:**
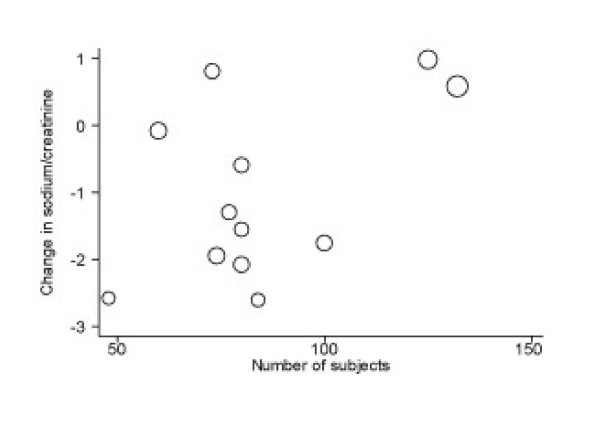
**Changes in sodium-to-creatinine ratio adjusted for age, sex, BMI at baseline and locality by number of subjects recruited from each village**. Regression coefficient = -0.03 [-0.05 to 0], p = 0.042 from meta-regression analysis.

The intervention and control groups were comparable for baseline characteristics (Table 2). In the intervention group 47% were from rural villages compared to 53% in the control group. During the health promotion programme the change in average sodium excretion varied between villages. It fell in four out of six villages in the intervention group and in 5 out of six villages in the control group (Figure 2). The net intervention effect was therefore a non-significant change in sodium excretion (Table 3 and 4). Smaller villages (with smaller samples) showed a greater reduction in urinary sodium excretion than larger villages (with larger samples) (p = 0.042) (Figure 3).

### Effect of the intervention programme on BP

#### Intention-to-treat analysis

Throughout the trial we observed that the intervention group showed a small reduction in both systolic and diastolic BP, more pronounced at 6 months and statistically significant for diastolic BP at 6 months (Table 3 and 4). This effect was not consistent with the effect observed on urinary sodium excretion (Table 3 and 4).

#### On-diet analysis

At both 3 and 6 months, there was a consistent and significant relationship between the fall in urinary sodium excretion (however expressed) and the fall in systolic BP, when adjusting for confounders (Table 5). A 50 mmol per day lower urinary sodium was associated with a fall in systolic BP between 1.0 and 3.2 mmHg at 3 months and 0.1 to 2.6 mmHg at 6 months. The effect on diastolic BP was less consistent, although in the same direction.

## Discussion

This is the first attempt of a community-based trial of health promotion in rural and semi-urban villages in sub-Saharan Africa to achieve a population-wide reduction in salt intake with a view to reducing the population BP. The programme was complex and beset by practical difficulties. It has nevertheless produced important findings.

We have arrived at estimates of BP and salt intake in the Ashanti region of Ghana, and have gathered data on the efficacy and potential effectiveness of a programme of a modest reduction in salt intake in reducing the BP of African villagers. We chose the most difficult setting to implement a population-wide strategy, i.e. rural communities where the people are predominantly non-hypertensive and whose salt intake is lower than that found in most western countries.

### Strengths and limitations of our study

To avoid potential confounding due to the localities we undertook stratification at cluster level. We also stratified at individual level in order to achieve the same age-gender structure of the invited sample of each village. Recruitment was carried out blind to avoid possible recruitment bias if the response rate proved to be low – as it was in some villages. The response rate was lower than expected for reasons unrelated to the intervention itself. There was a delay of up to 18 months between the household survey and starting the fieldwork in some villages. Villagers had sometimes forgotten that they had agreed to take part in the study and others had died or moved out of the area. Blood sampling was also sometimes a cause of difficulty. Blood was taken to measure factors related to cardiovascular disease but in some villages rumours spread that the blood was to be tested for HIV infection; in Ghana as in other parts of the world a positive HIV test carries a stigma for the whole family. Another rumour was that the blood would be sold in London. The timing of the blood sample required thought. While the time of data collection (6 am onwards) suited those working on their farms locally, as well as those remaining in the village, it did not suit those who left for work much earlier. This phenomenon probably accounted for the lower response rate amongst men in semi-urban villages.

### Interpretation of our study

Despite our careful planning, we did not manage to complete a fully randomised trial due to the contamination of the two intervention arms. Initially, we found the intervention effects difficult to interpret since the changes in BP and sodium excretion within each village were less than expected from the pilot study [17]. Possible explanations included contamination due to two reasons. First, the participants of intervention and control villages could have had contact with each other and exchanged information about the content of the health promotion. This was unlikely as the villages had been selected because not only were they several kilometres away from each other but also used different markets, and had no easy means of communication [24]. Alternatively, contamination could have arisen through the health workers who, despite their training and awareness of the purpose and scope of the study and the importance of concealing the true objectives, did unwittingly give information on salt reduction to villagers in the control villages in the belief that they should attempt to improve the health of all the villagers. Finally, a true variation in response between villages cannot be excluded. The disappointing outcomes could have been related to differing attitudes of different fieldworkers, variable co-operation of villagers (some villages were united, some less so), proportion of villagers in the village at the time of the educational sessions, and clashes with other events related to farming.

If we accept that there was contamination – with some villages in the control group receiving advice on salt reduction, the regression analysis could provide an estimate of effect on BP of a reduction in urinary sodium excretion in all villages, irrespective of treatment allocation. We would then expect a greater reduction in BP for a greater reduction in urinary sodium. This is what we found, and it supports our concept. It can be argued, however, that this association can be merely due to some changes over time having an effect on both sodium and blood pressure. We cannot rule this out. We do not think our findings are explained by chance, though.

When measuring BP, the possibility of confounding factors at village level cannot be ruled out, and we have shown, for example, that time of day is a significant predictor of BP. Since we have no records of ambient temperature, we have had to use time of day instead. The difference in BP predicted by a 2 h difference in the time of day is of the same order of magnitude of that expected by the intervention. Since all measurements were taken on the same day in each village, the BP measurements are also highly clustered. This means that in some villages, because of the effect of temperature, the BP may have risen even though sodium excretion may have fallen.

### Our data in the context of Africa

Two intervention trials of dietary salt reduction in populations of African origin have been published so far [18,19]. Both were carried out in clinical settings under controlled experimental conditions and lasted 3-weeks. The authors suggest that the efficacy of a reduction in salt intake in developing countries equals that noted in more affluent cultures and in whites [16,29]. Our study extends these observations for the first time to a community setting and on a longer term to provide some evidence of effectiveness. As expected, the longer the study – in our case health promotion – the less pronounced the efficacy, presumably as a result of adaptation and reduced compliance as has been shown in studies in the Western world dealing with salt intake [30] and weight reduction [31].

Some have argued that health promotion programmes of cardiovascular prevention in the Western world should not be exported to developing countries because such programmes would be difficult to implement [32,33]. They have suggested that '*counting deaths*' – a relatively easy task that focuses on 'surveillance' of cardiovascular disease in Africa – would lead to more effective prevention [32]. At the same time, they recognise that '*it is essential that context-appropriate health research and health interventions take place in developing countries*' [33].

It would be simplistic to pre-judge the outcome of context-specific health promotion strategies based on experiences of the western world. Cultural and behavioural factors are often responsible for successes or failures of community interventions and it is likely that different mechanisms would operate in different contexts, so defeating mere generalisations. Furthermore, educational interventions in whole communities are 'complex interventions' that often pose significant problems of evaluation due to the difficulty of standardising their components [34].

Within the context of low-income countries in epidemiological transition, the case in favour of a population-based approach to limiting the rise in BP and ensuing stroke and renal risk is compelling [35,36]. In Ghana, structures for the detection, management and control of high BP outside cities and large towns are virtually absent [6]. Anti-hypertensive drugs are not free and most people in need cannot afford them [37]. It has been argued that drug treatment of cardiovascular risk factors in African communities will divert the already scarce resources away from needier areas of healthcare, such as maternal and child health, diarrhoeal diseases and malaria. The advent of free anti-retroviral therapy in many areas of sub-Saharan Africa will necessarily lead to the development of systems for the provision of drugs for the management of other chronic diseases. However, such advances are unlikely for many years, and the cheaper option of encouraging lifestyle changes is likely to be the preferred policy for some time.

### Perspectives

In the DASH-Sodium study a reduction from 6.3 to 3.9 g of salt per day (~38 mmol of sodium per day) in non-hypertensive people caused a reduction in BP of 3.4/2.0 mmHg [38]. The effect was much greater in hypertensives, in African Americans and more pronounced the greater the change in salt intake [16,38]. By using the combined evidence from trials in the literature a modest reduction in salt intake of ~3 g per day (50 mmol of sodium) in non-hypertensive people would reduce BP by 2 to 4/1 to 2 mmHg [39]. This reduction, if sustained long-term, would be responsible for a reduction in stroke events of 12–14% per year [29,39]. The efficacy of salt reduction in lowering BP levels has been confirmed recently in two small short-term clinical trials in Nigeria [18] and Jamaica [19].

How far would a reduction of 1.3 mmHg in population systolic BP – as shown in our study – impact on stroke rates? No randomised clinical trials have been performed to show that a sustained reduction in salt intake leads to a reduction of cardiovascular events; however, evidence from prospective studies suggests that those eating less salt have a reduced risk of cardiovascular events [21,40-42]. From prospective studies, randomised clinical trials of BP lowering treatment with drugs and short term trials of salt restriction it is estimated that stroke rates would fall by at least 10% in non-hypertensive individuals whose salt intake was reduced by 50 mmol per day and systolic BP by 1.3 mmHg [39]. In hypertensive individuals the benefit would be much greater. There is no evidence so far to guide us to extrapolate these benefits to African populations. It can be argued, however, that the attributable preventive fraction could be greater, given less competing cardiovascular risk in these populations.

Finally, our study design aimed specifically at changing salt intake, without targeting other aspects of the participants' diet. It is conceivable that a combined approach with an increase in potassium intake (not as high in this population as one would have expected) alongside a modification of their fat intake (mainly coming from the use of palm and coconut oils rather than dairy products) might exert compounding effects on lowering of blood pressure and prevention of strokes.

## Conclusion

The impending epidemic of cardiovascular disease in sub-Saharan Africa in a context of lack of resources is a serious global public health challenge. Community-based strategies of health promotion for the management of chronic disease through lifestyle change in sub-Saharan Africa should be considered.

## Competing interests

The author(s) declare that they have no competing interests.

## Authors' contributions

FPC, SMK, JP-R and JBE contributed to fund raising, idea for and design of the study, collection of data, interpretation of results. FPC and SMK drafted the paper. FBM contributed to collection of data and interpretation of results. All authors contributed to the critical revision of the paper. FPC had full access to all the data in the study and takes responsibility for the integrity of the data and the accuracy of the data analysis

## Pre-publication history

The pre-publication history for this paper can be accessed here:



## Figures and Tables

**Table 1:** Regression coefficients (and 95% C.I.) for blood pressure^¶ ^over sodium excretion adjusted for age, sex, locality, body mass index (random effect model)

**Unit change**	**Systolic B.P. (mmHg)**	**Diastolic B.P. (mmHg)**
50 mmol of 24 h urinary sodium	2.17 (0.44 to 3.91)^#^	1.01 (0.08 to 1.94)^#^
Unit of sodium: creatinine ratio	0.45 (0.17 to 0.75)^#^	0.18 (0.02 to 0.33)^#^
Unit of sodium: potassium ratio	1.74 (0.45 to 3.03)^#^	1.16 (0.43 to 1.80)^#^

^¶ ^Adjusted for time of day. ^# ^p < 0.001

**Table 2:** Baseline characteristics of the population in the intervention and control groups.

	**Intervention (n = 522)**	**Control (n = 491)**
Age	54 (11)	55 (11)
Female n (%)	324 (62)	304 (62)
Height in cm	160 (8)	161 (8)
Weight in kg	54 (11)	54 (11)
Body mass index (kg/m^2^)	21 (4)	21 (4)
Systolic blood pressure (mmHg)^¶^	129 (25)	127 (27)
Diastolic blood pressure (mm Hg)^¶^	77 (13)	76 (13)
Heart rate (bpm)^¶^	74 (12)	72 (12)
Urinary sodium (mmol/24 h)	99.9 (44.7)	102.5 (45.3)
Urinary potassium (mmol/24 h)	47.5 (23.2)	49.3 (23.6)
Urinary creatinine (mmol/24 h)	8.4 (2.6)	8.4 (2.6)
Urinary sodium/potassium ratio	2.4 (1.2)	2.4 (1.3)
Urinary sodium/creatinine ratio	12.4 (5.3)	12.7 (5.6)
Urinary potassium/creatinine ratio	5.9 (2.8)	6.1 (2.8)
Current smoking n (%)	31 (6)	41 (8)
Alcohol drinking n (%)	199 (38)	211 (43)
Hypertensive n (%)	154 (30)	137 (28)

2 subjects have missing urine collections at baseline

^¶ ^Adjusted for time of day

Results are means (standard deviations) or number (percentage)

**Table 3:** Baseline and (a) three months values or (b) six months values for blood pressure^¶ ^and sodium and potassium excretion for all participants followed-up to that time point.

(a)	Baseline		Three months	
	
	Intervention (n = 444)	Control (n = 450)	Intervention (n = 444)	Control (n = 450)
Systolic blood pressure (mm Hg)	128.5 (24.7)	127.3 (26.3)	124.6 (26.6)	123.8 (26.0)
Diastolic blood pressure (mm Hg)	76.8 (13.0)	75.8 (13.5)	74.2 (13.7)	74.0 (14.1)
Urinary sodium (mm/24 h)	100.9 (44.3)	103.6 (45.3)	94.0 (44.5)	97.5 (42.3)
Urinary potassium (mm/24 h)	48.3 (23.5)	49.3 (23.4)	50.1 (20.8)	52.1 (23.6)
Urinary creatinine (mm/24 h)	8.4 (2.6)	8.4 (2.6)	7.8 (2.5)	7.9 (2.8)
Urinary sodium/potassium ratio	2.4 (1.2)	2.4 (1.3)	2.1 (1.1)	2.1 (1.1)
Urinary sodium/creatinine ratio	12.4 (5.2)	12.8 (5.6)	12.4 (5.4)	13.0 (5.6)
Urinary potassium/creatinine ratio	6.0 (2.8)	6.1 (2.8)	6.7 (2.9)	6.9 (2.9)

(b)	Baseline		Six months	
	
	Intervention (n = 399)	Control (n = 402)	Intervention (n = 399)	Control (n = 402)

Systolic blood pressure (mm Hg)	129.2 (24.6)	125.6 (25.5)	127.9 (27.7)	127.4 (26.0)
Diastolic blood pressure (mm Hg)	76.9 (13.0)	75.2 (13.3)	76.0 (14.2)	78.7 (14.3)
Urinary sodium (mm/24 h)	100.7 (45.2)	104.2 (45.5)	91.8 (41.8)	89.8 (39.1)
Urinary potassium (mm/24 h)	48.5 (23.9)	50.9 (24.2)	48.2 (21.5)	52.1 (21.9)
Urinary creatinine (mm/24 h)	8.4 (2.6)	8.4 (2.5)	8.0 (2.6)	8.0 (2.5)
Urinary sodium/potassium ratio	2.4 (1.3)	2.4 (1.3)	2.2 (1.2)	1.9 (1.0)
Urinary sodium/creatinine ratio	12.4 (5.2)	12.8 (5.6)	11.9 (5.1)	11.7 (4.7)
Urinary potassium/creatinine ratio	6.0 (2.9)	6.3 (2.9)	6.3 (2.7)	6.8 (2.7)

^¶ ^Adjusted for time of day

Missing values for urinary variables; intervention, 3 months n = 1, control, baseline n = 1, 3 months n = 4

Missing values for urinary variables; control, baseline n = 1, 6 months n = 1

**Table 4:** Effect of intervention (and 95% CI) on reduction in blood pressure^¶ ^and urinary sodium excretion at 3 and 6 months adjusted for age, sex, locality, body mass index (random effect model)

**Variable**	**3 months (n = 894)**	**6 months (n = 801)**
SBP (mmHg)	-0.48 (-5.45 to 4.50)	-2.54 (-6.54 to 1.45)
DBP (mmHg)	-1.02 (-3.95 to 1.91)	-3.95 (-7.11 to -0.78)**
Urinary sodium (mmol/24 h)	-0.5 (-12.3 to 11.3)	6.0 (-4.1 to 16.1)
Urinary sodium: creatinine	-0.01 (-1.35 to 1.32)	0.85 (-0.53 to 2.24)
Urinary sodium: potassium	0.08 (-0.22 to 0.39)	0.30 (0.02 to 0.57)*

^¶ ^Adjusted for time of day *p = 0.03, **p = 0.015

Effect is control – intervention (negative values favour intervention).

**Table 5:** Regression coefficients (and 95% C.I.) for changes in blood pressure^¶ ^for a given change in sodium excretion adjusted for age, sex, locality, body mass index (random effect model)

**Unit change**	**Month**	**Systolic B.P. (mmHg)**	**Diastolic B.P. (mmHg)**
50 mmol of 24 h urinary sodium	3	2.12 (1.03 to 3.21)^#^	1.11 (0.45 to 1.78)^#^
	6	1.34 (0.08 to 2.60)*	0.30 (-0.45 to 1.04)
Unit of sodium: creatinine ratio	3	0.33 (0.15 to 0.51)^#^	0.14 (0.03 to 0.25)**
	6	0.30 (0.08 to 0.53)^§^	0.03 (-0.10 to 0.17)
Unit of sodium: potassium ratio	3	1.59 (0.73 to 2.46)^#^	0.48 (-0.05 to 1.00)
	6	1.94 (0.97 to 2.90)^#^	0.47 (-0.11 to 1.04)

^¶ ^Adjusted for time of day ^#^p < 0.001; ^§^p = 0.008; **p = 0.015; *p = 0.037
